# Untangling the Governance of Public Health Aspects of Manure in The Netherlands

**DOI:** 10.3390/ijerph182312472

**Published:** 2021-11-26

**Authors:** Sophia Dollmann, Lucie Vermeulen, Ana Maria de Roda Husman

**Affiliations:** 1 Centre for Infectious Disease Control, National Institute for Public Health and the Environment (RIVM), Antonie van Leeuwenhoeklaan 9, 3721 MA Bilthoven, The Netherlands; lucie.vermeulen@rivm.nl (L.V.); ana.maria.de.roda.husman@rivm.nl (A.M.d.R.H.); 2 Institute for Risk Assessment Sciences (IRAS), Utrecht University, Yalelaan 2, 3584 CM Utrecht, The Netherlands

**Keywords:** agricultural policy, manure treatment, export, risk management, risk communication, system mapping

## Abstract

The Netherlands is one of the most densely populated countries in terms of people and livestock and is the second largest exporter of agricultural products worldwide. As a result, the Netherlands has a manure surplus. Excess application of manure can lead to environmental problems; therefore, manure needs to be treated and discharged. Manure can contain zoonotic pathogens, but whether exposure to manure and manure treatment also poses a risk to public health is still unknown. This study analysed the regulations, relevant actors, and responsibilities in the complex system of manure and public health in the Netherlands. Interviews and system mapping have demonstrated interlinkages between environmental, economic, and health aspects. Constraints and opportunities for public health protection have been identified. This study reveals the complexity of the Dutch manure policy, its scattered responsibilities, the challenge to deal with uncertainties, and, most importantly, the need for a microbial risk assessment in order to adequately communicate and manage possible risks to protect the health of animals, the environment, and people.

## 1. Introduction

The Netherlands is characterized by a high population, as well as high livestock densities [1]. As animal products represent some of the main export products, one of their side products, manure, is also being produced in great amounts [2]. The majority of fresh manure stays in the country and needs to be managed locally, because it is not considered as an essential export product [3]. Over 70% of the total Dutch manure is directly applied on fields close to the location where it was produced and serves as an organic fertilizer for grassland or crops [4]. However, the available nutrients from manure exceed the total requirements needed by grasslands and crops. This manure surplus could harm the living environment. In particular, the release of excess nutrients from manure can deteriorate surface water quality, ground water quality, and can cause eutrophication, which impairs terrestrial and aquatic ecosystems, as well as animal health [5]. Manure can be a source of antimicrobial resistance, particulate matter, and endotoxins [6,7]. Additionally, several studies have detected pathogens in manure, which might reach surface water or air after soil application [8,9,10]. These pathogens are often zoonotic and can cause severe disease when transmitted to humans, whilst being asymptomatic in animals [11]. In the past decades, manure-borne pathogens have caused outbreaks, such as the Dutch Q-fever epidemic in 2007 and a Shiga toxin-producing *Escherichia coli* (STEC) outbreak in the United States [12]. Consequently, there is increasing concern among residents of livestock dense areas, who have initiated the discussion about the effects of livestock farming and manure on public health [13,14,15].

Since manure could potentially pose a risk to public health, it is important to understand the governance of manure from a public health perspective. Governance, as it is defined by van Asselt and Renn [16], is the ensemble of actors and processes which result in binding decisions. Visualizing the governance structures in a system map might indicate constraints and opportunities to protect public health, alongside the other objectives of Dutch manure policies.

Therefore, this paper aimed to reveal how public health aspects are governed regarding Dutch manure policies. By means of a system map, this study aimed to identify the relevant actors, acts, and processes which are related to the public health risks of manure in the Netherlands. We hypothesized that system mapping could demonstrate gaps and opportunities in the current manure policy, with regards to public health protection. In the Discussion, constraints and opportunities are identified in relation to the public health risks caused by manure.

## 2. Materials and Methods

Based on the literature and input from interviews, the system map was developed. Systems, as defined by Arnold and Wade [17], are groups or combinations of interrelated, interdependent, or interacting elements forming collective entities. According to Gopal et al. [18], system mapping addresses problems that cannot be solved on an individual level [18]. Therefore, “system maps are powerful visualization tools that can help change agents, describe and diagnose the current state of a given system and identify opportunities for improvement” [18]. In addition, the purpose of a system map is to recognize gaps in order to strengthen the governance system [18].

To start designing a system map, its boundaries need to be defined. The boundaries of the Dutch manure policies and public health regulations were set by mapping the EU-wide and national regulations that are associated with manure, as well as the established players. Moreover, the Driver-Pressure-State-Impact-Response (DPSIR) framework was used to structure the system map [19]. This framework can be used to visualize the human impact on the environment [19]. Another reason for this model choice is based on its possibility to display complex interconnections between anthropogenic activities and their effects on the environment and society [20]. In this paper, the DPSIR framework was adapted to also consider public health aspects.

Experts within the field were consulted to identify relevant actors who were familiar with manure governance. Applying a form of snowball sampling, some subjects were chosen via recommendations from former interviewees. This resulted in seven interviewees from different organizations and roles in the system of manure and health (Table 1). The interviews, which were conducted face-to-face between August and October 2018, lasted approximately sixty minutes and were recorded in order to be later transcribed. The subjects all signed an informed consent form. The interviews on the position of health risks and manure were semi-structured, and followed a list of questions (Appendix A). The interviews were structured based on questions about their work and its relation to manure and public health, their knowledge and perspectives about possible public health risks and manure policies, as well as their existing knowledge and opinions on policy gaps. As a consequent step, the transcripts were coded and analysed using the MAXQDA^®®^ software (VERBI Software GmbH, Berlin, Germany), as explained in Kuckartz and Kuckartz [21]. This analysis tool helps in coding segments of the transcribed interviews to analyse the overall picture.

The last step included the revision and refining of the maps by incorporating the interviewee’s suggestions. Since different experts were consulted, the system map was adjusted after every interview. Their input was used to identify the key actors and their roles in the system. Additionally, existing regulations and new developments were included in the system map. Furthermore, the input from the interviewees, with different responsibilities and perspectives on this topic, helped to identify implications, blockages, and opportunities. Seven players were interviewed. Since the concepts that were discussed during these seven interviews occurred repeatedly, this might have already suggested data saturation. The system map could still capture a more integral view if the number and nature of interviewees was extended. In the case of addressing the suggestions from the interviewees, these are covered in the Discussion.

## 3. Results

### 3.1. Processes, Policies and Actors along the DPSIR Framework

**Figure 1:**
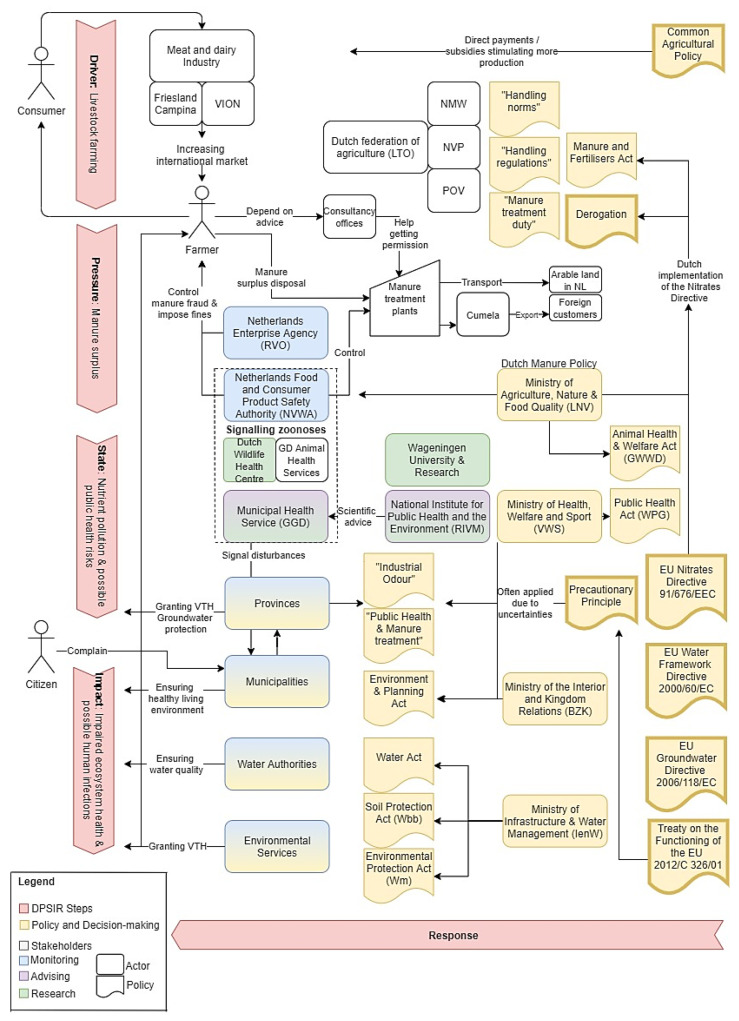
System map of manure and public health in the Netherlands. The arrow representing the responses is depicted horizontally and can exert influence on every step of the framework. NMV (Nederlandse Melkveehouders Vakbond): Dutch dairy farmer union; NVP (Nederlandse Vakbond Pluimveehouders): Dutch trade union for poultry farmers; POV (Producenten Organisatie Varkenshouderij): association of pig farmers; VTH (Vergunningverlening, Toezicht en Handhaving): authorization, monitoring and enforcement.

Based on data from the literature and the interviews, the system mapping approach has resulted in an overview of the governance of public health aspects of manure in the Netherlands. The system map illustrates the regulations and actors as well as their roles and responsibilities in relation to manure and public health protection, alongside the DPSIR framework. The steps identified, from the drivers to the impact, are illustrated vertically, starting at the top of Figure 1. All measures that were taken to prevent pollution and public health risks in this system are part of the responses. The respective elements of the DPSIR analysis are described one by one.

#### 3.1.1. Driver: Livestock Farming

The governance of manure, as presented in Figure 1 and as analysed in this study, is driven by livestock farming. This sector is represented by the Dutch Federation of Agriculture and Horticulture (LTO), under which there are more specialized associations, such as the Dutch dairy farmer union (NMV), the Dutch trade union for poultry farmers (NVP), and the association of pig farmers (POV), which each represent their own sub sector. Not only Dutch consumers, but especially the international market, demand dairy and meat products, as 67% of Dutch pigs and pig meat are exported [22]. Friesland Campina and VION are two of the largest companies in the dairy and meat industry. This type of industry and its size are stimulated by European regulations, such as the Common Agricultural Policy (CAP), which subsidizes mainly meat, dairy, and animal food [23]. Moreover, farmers react to the low prices for animal products by upscaling their farms [24].

#### 3.1.2. Pressure: Manure Surplus

The majority of Dutch livestock in 2021 consists of 3.7 million cattle, 11.5 million pigs, and 90 million chickens [25]. This magnitude of livestock farming puts pressure on the system of manure production and use, as well as its consequences for environmental and public health. “70% of this animal production is meant for export, not for the Dutch consumer. But we have to face the problems caused by a sector that produces mainly for the export” (Interviewee 1). These problems imply approximately an annual production of 75 million tonnes of manure [26], which exceeds the national nutrient targets [1]. Manure from both livestock in stables, as well as on pasture, contribute to the Dutch manure surplus. Dairy cattle account for 80% of the manure production, followed by pigs (13%). This concentrates in the province North Brabant, which exceeds the average number of livestock per farm. In 2021, the average number of dairy cows on a Dutch farm per hectare is 1.77. In North Brabant, one hectare of arable land holds 2.21 cows. The same trend holds true for pig farms in the Netherlands, which own, on average, 3400 animals. In North Brabant, this number is 4800 [27].

#### 3.1.3. State: Nutrient Pollution and Possible Public Health Risks

##### Manure Regulations

Manure volumes are regulated within the Manure and Fertilizers Act, which is the Dutch implementation of the EU Nitrate Directive (91/676/EEC) in order to prevent the nutrient pollution of ecosystems. On top of the general restrictions, the Netherlands has obtained a locally applicable adaptation to the Nitrate Directive, called “Derogation”. It allows farmers with arable land to apply, annually, up to 250 kg instead of 170 kg of manure per hectare of land, as long as this does not impair the shallow groundwater quality. Farmers need to fulfil certain conditions, such as the monitoring of environmental effects, having a minimum percentage of permanent grassland compared to cropland, and having to apply for this permit at the Netherlands Enterprise Agency (RVO). The norms that come with the Derogation entail the handling norms, handling instructions, and the duty of manure treatment [28]. These also aim to prevent odour nuisance for residents. The handling instructions indicate how farmers need to handle their manure. The handling norms determine the amount of manure that can be applied. Farmers with cattle on pasture need to apply the phosphate rights, which specify the number of animals allowed and the amount of phosphate they are allowed to produce. This calculation is based on the size of the pasture area, the type of soil and crops, the milk production and the relative phosphate excretion. A farmer needs to have a sufficient area of arable land for the number of dairy cattle they own to dispose the produced manure on their own land. For pig and poultry farmers, the production rights hold. The production rights also restrict the farmer to a maximum allowed number of animals, in order to control the amount of produced manure. Mostly, pig farmers do not own much arable land where they could dispose their produced manure, and are therefore obliged to treat a percentage of this manure surplus. Manure treatment entails, for instance, fermentation in biogas plants, digestion, hygienisation, composting, separation, or reverse osmosis. The main advantage of manure treatment is the prevention of nutrient losses. Additionally, manure treatment reduces the manure’s water content. This results in a mineral concentrate which makes the transport less costly. Manure products can be transported from manure-surplus regions to regions where manure is needed, to provide nutrients to crops [29,30].

##### Manure Treatment

According to the Derogation, excess manure needs to be transported to other plots of arable land within 20 km of the farm or be treated. Before export to other European countries, manure must undergo a specific treatment, which is called hygienisation [31,32]. Hygienisation entails the heating of manure to at least 70 °C degrees for one hour [33,34]. “The compulsory treatment of manure can already limit the microbiological risks to a great extent” (Interviewee 5). However, the underlying reason to treat manure is not related to public health protection, (…), but to the spread of animal diseases to agricultural areas abroad, which could interfere with livestock production. It is not a public health aspect, maybe a sort of positive side effect.” (Interviewee 1). Interviewee 3 supports this statement: “There are possible public health risks of manure, but the regulations on the handling and the storage of manure are not arranged for public health aspects. However, they certainly have an influence on public health.” The downside of this obligation is that “disposing manure costs the average Dutch farmer, on average, 45,000 euro annually. The Dutch Ministry of Agriculture, Nature and Food Quality (LNV) stimulates manure treatment plants with subsidies in order to increase the treatment capacity [35]. But farmers do not receive any financial support for the handling procedures with manure. These costs are so high that manure fraud indeed becomes appealing to the farmer.” (Interviewee 1). These aspects provide insight into the farmer’s position. In particular, pig farmers carry an increased burden. Besides the fact that their business is not lucrative, as pig meat is offered at a low price, pig farmers without entry to sufficient areas of agricultural land are required to manage the time- and money-consuming manure disposal.

The handling of manure is controlled by the Netherlands Food and Consumer Product Safety Authority (NVWA) and the Netherlands Enterprise Agency (RVO), who determine whether farmers comply with their duty to treat manure, as well as whether manure transport is properly managed. “The NVWA is the controlling party of the Fertilizers Act.” (Interviewee 1). In case they detect manure fraud, farmers will be fined. “Farmers are trapped in these structures”, as Interviewee 1 stated, and often lose sight of the rules and regulations they have to adhere to. Hence, they ask consultancy offices for advice in handling the complicated manure policies. Unfortunately, this advice is not always independent, as it also comes with other specific recommendations, e.g., regarding livestock feed. Furthermore, consultancy offices can help farmers in starting a new farm or manure treatment plant by preparing the application forms for permission from the licensing authorities, which are often a province or municipality. Entrepreneurs of manure treatment plants have to fulfil certain conditions, which are stated in the manure policy and the so-called activities decree. These aim to ensure the safety of the manure treatment plants regarding their effect on the environment. The incorporation of these terms must be presented in the license application. “Without the advisors, the farmers would not get the license to treat manure. This would only lead to an increase of the illegal manure handling” (Interviewee 5).

##### Manure Treatment Plants and Local Residents

The authority’s decisions to grant licences for manure treatment initiatives are public. Thus, citizens, of whom also includes those living in the vicinity of manure treatment plants, can become involved. Residents who worry about noise, odour nuisance or their health can lodge an objection, which complicates the process of launching new manure treatment plants. “As there are still a lot of uncertainties on the risks of manure and manure treatment on public health, people regard themselves as guinea pigs.(…) The Q fever outbreak in 2007 has resulted in a traumatized resident population that does not trust the government nor science.” (Interviewee 4). The “not in my backyard” concept applies here very well, as residents do not want any manure or manure treatment nearby. It has become a polarized discussion, since people living in livestock-dense areas only want a reduction of livestock animals and, consequently, less manure. As reported by an interviewee, residents use their access to justice and protest against these initiatives, which hinders the treatment of excess manure and impairs the situation even more. “One manure treatment plant was supposed to be built in a rural area but has been impeded by the protest of residents. After long discussions, the Province of North Brabant has decided to place this facility in an industrial area and again there was protest. The entrepreneurs are concerned with this facility since ten years and it is still not working” (Interviewee 6). “It is about the resident’s feeling. Their expressed concern is actually based on their opposition to the amount of livestock animals” (Interviewee 6). According to Interviewee 3, farmers often lack societal consensus and public support, broadening the gap between residents and farmers. Another underlying reason for their distrust and concern might be due to problems in the communication between the livestock industry and residents, as well as uncertainties concerning microbiological emissions from manure treatment plants [13,36].

##### Manure and Public Health

The current Dutch manure policy does not cover the microbiological risks for public health, but instead focuses on the broader policy goals of ensuring a healthy environment, as well as the improvement and maintenance of good water quality and the prevention of eutrophication [37]. “There are several regulatory measures that partially consider public health aspects, as water quality is associated with public health. The surface water and groundwater quality needs to guarantee clean and safe water, which can be used for recreation or for drinking water for livestock” (Interviewee 3). Environmental aspects are the main responsibilities of the Ministry of Infrastructure and Water Management (IenW). They collaborate with the Ministry of Agriculture, Nature and Food Quality (LNV). “We (team manure of LNV) implement the Nitrate Directive which also needs to contribute to the Water Framework Directive. These are both EU directives.” (Interviewee 3). Moreover, the Groundwater Directive aims to “achieve water quality levels that do not give rise to significant impacts on, and risks to human health and the environment” [38]. In addition, the Dutch Water Act governs surface and groundwater quality by chemical and ecological standards. “For water quality standards, the Water Act refers to lists of substances and standards provided by the Environmental Management Act and the Groundwater Directive” [39]. The Soil Protection Act regulates that soil “ […] does not pose any unacceptable risks to public health and does not pose a threat to the functional properties of water, soil and air for people, plants and animals” [40]. The role of these municipalities is to ensure a healthy living environment. Dutch water authorities regulate safe water quality. In addition, provinces take charge of groundwater protection areas, where water is extracted for drinking water production. These are monitored by the means of employing critical values for concentrations of nutrients or of other substances from agriculture.

#### 3.1.4. Impact: Impaired Ecosystem Health and Possible Human Infections

The underlying reasons for manure fraud have been described in an earlier Section 3.1.3. state. Manure fraud refers to documenting the removal of manure to other destinations, but in fact applying that manure on the land. This can cause nutrient runoff, which impairs the quality of ground water or surface water, with eutrophication and biodiversity loss as possible outcomes. Excess nutrients can promote algal blooms, which produce toxins that are harmful for animal and human health [41,42].

Regarding the risks on public health in terms of infectious diseases, the impact cannot be defined, as little to no infectious disease outbreaks in the past have been linked to manure. Manure, according to van den Brom, et al. [43], only played a minor role in the Q fever outbreak in 2007–2010, which affected people living close to goat farms. Other studies, however, showed that contact with manure or those who live close to the distribution of manure was associated with a higher risk of Q fever infection [44,45]. Still, many uncertainties on the public health risks from manure and manure treatment remain [3,46]. However, surrounding residents of intensive livestock production facilities and manure treatment plants may suffer from odour nuisance, which impacts their wellbeing [47]. Perceived odour nuisance increases residents’ irritation and health concerns, due to the uncertain public health risks of manure. Moreover, manure could spread antimicrobial resistance to the soil and surface water [6]. Nearby residents of intensive livestock production have also been shown to be carriers of antibiotic-resistant bacteria, such as Methicillin-resistant *Staphylococcus aureus* (MRSA), to a greater extent [7]. In addition, they are more susceptible to respiratory infections, due to higher endotoxin and ammonia concentrations in the air. Manure is a source of ammonia and endotoxins, which can impair lung function. This results in higher risks for pneumonia and increased problems among COPD patients who live close to intensive livestock farms [7]. Additionally, new zoonotic disease risks might emerge [48], which can be introduced into the environment via manure.

#### 3.1.5. Response: Environmental Protection Policy, Surveillance of Zoonoses

The responses to each step (drivers, pressure, state, impact) of the system map (Figure 1), with regards to public health either indirectly entail the environmental protection or achieve this directly, through the reduction of odour nuisance as well as through the signalling of zoonoses which could pose a threat to public health. Below, some examples of responses related to the modules of Figure 1 are outlined.

##### Authorization, Monitoring, and Enforcement

The provinces are primarily involved in environmental planning, which has to consider public health protection. “The municipalities, however, need to be in accordance, by stating they have no objections” (Interviewee 4). Thus, the authorities granting permits, and who monitor and enforce regulations can ensure that manure handling is done in the best possible way, to reduce the risks to public health and the environment. “You can ensure a healthy living environment by providing permits that are bound to strict but clear instructions and requirements, which can actually be fulfilled by the entrepreneur.” (Interviewee 4). The Environmental services are in charge of authorization, and carry out the surveillance and monitoring of compliance with the regulations that come with permits, such as emission limits. Manure treatment plants, for instance, require an air scrubber to reduce emissions and, hence, odour nuisance. “Without adequate monitoring, the occurrence of emissions increases” (Interviewee 4). Authorization, monitoring, and enforcement duties include that the licenses are bound to terms, which provides a safe framework that entrepreneurs have to fulfil. The control and enforcement entails that these have to be checked, and fulfilled, if something has deviated from the agreement.

##### Environment and Planning Act

The future Dutch Environment and Planning Act (expected to be enforced in 2022), involved in the combining and integrating of a large set of separated, thematically oriented acts, might provide new opportunities to protect public health with regard to manure. In the case of manure being proven to be a potential risk factor for public health, this Act could therefore facilitate the implementation of specific interventions, reducing the microbiological risks of manure for public health. The authority in charge for implementing the Environment and Planning Act is the Ministry of the Interior and Kingdom Relations (BZK), as well as, partially, the Ministry of Public Health, Welfare, and Sports (VWS). “In some areas, where we think that health is insufficiently covered, we have to intervene. For example, health is now mentioned in the new Environment and Planning Act.”(Interviewee 3). This covers the spatial distribution of industrial areas, urban areas, and the environmental impacts from industry, whereas manure only plays a minor role. It is still unclear how manure and health can be covered in the Environment and Planning Act. “Therefore, a health impact assessment of manure handling and manure treatment plants on environmental and public health should be done” (Interviewee 2). The Province of North Brabant wants the precautionary principle to be incorporated in the new Environment and Planning Act as this “… could make it harder for entrepreneurs to cause emissions.” (Interviewee 6). Another interviewee stressed the importance of assessing permit applications. “The Environment and Planning Act can certainly provide a connecting factor, as long as health is included. It is important to have a proper evaluation framework, which takes into account all public health protection aspects. For instance, the hygienisation of manure would already solve the microbiological problem for the most part.” (Interviewee 5). Interviewee 7 did not think that the Environment and Planning Act could contribute much more than the current planning permission already does. “Possible risks for public health have to be assessed. Incidentally also for farmers as they are even more exposed than the residents. It is simply part of the Environment and Planning Act that you need a permit that safeguards the health of the residents.” (Interviewee 7).

##### Signalling Health Risks

Intensive livestock production, including manure, also has an impact on local residents. Their concerns and complaints rest upon the odour nuisance which they perceive. Odour might be an indicator for emissions, such as for particulate matter that can impair public health. The Municipal Health Service (GGD) does not only consider health impacts that are caused by microorganisms, but also by “odour, noise, particulate matter, dust, and endotoxins” (Interviewee 4). They concentrate on the dialogue with residents, as Interviewee 5 supported: “It is important to communicate risks to the residents in an adequate way”. Additional responses by the GGD include conducting research and giving advice to municipalities, as well as signalling undesired situations or disturbances. To this end, the GGD also cooperates with the National Institute for Public Health and the Environment (RIVM) who work on behalf of the Ministry of VWS, as well as with other research institutes, such as Wageningen University & Research. They can be asked for scientific advice in order to support policy making or the communication of best practices. The GGDs are also part of the Dutch signalling forum for zoonoses (Signaleringsoverleg Zoonosen SO-Z). This group consists of experts with different orientations. The GGD receives signals on human health, whereas the GD Animal Health Service, NVWA, and the Dutch Wildlife Health Centre have information on the state of animals’ health. This signalling structure for zoonoses convenes on a monthly basis. Professionals from the food, wildlife, veterinary, and medical fields gather to discuss any odd observations that might need to be investigated. “In this way, we try to detect zoonoses before things get out of hand.” (Interviewee 2). In the case of these meeting discovering a signal of zoonotic infection that may pose a risk to public health, it will be further communicated for assessment and consequent action.

Moreover, the Animal Health and Welfare Act (GWWD 1992) governs the protection of animal health and well-being, which includes the prevention of animal diseases, which can be zoonotic and also pose a risk to human health. Hence, the GWWD could also protect public health. Interviewee 3 said that “we can ride free on other policy areas that cover health issues as a side effect“. In the case that the GWWD does not enable measures and the Ministry of VWS thinks that measures need to be taken, then the municipality can take action based on the Public Health Act (WPG). However, the Animal Health and Welfare Act is the *Lex specialis*, meaning that the law that is most attributed to govern the issue is the law that applies, when two laws relate to the same subject matter [49]. “You want to avoid that two laws govern the same issues. That is the reason why the Public Health Act does not include the topics that are already covered in the Animal Health and Welfare Act. In the case that something is not regulated in the Animal Health and Welfare Act, it can be done based on the Public Health Act. Hence, the Public Health Act is a kind of safety net.” (Interviewee 2).

##### Health in All Policies

Since the Dutch livestock production is mainly concentrated in the province North Brabant, it also has its own provincial ordinances on “Public Health and Manure Treatment” and on “Industrial Odour”. The provincial ordinance on Industrial Odour describes how odour needs to be handled. A paragraph on manure treatment is also incorporated [50]. The provincial ordinance on Public Health and Manure Treatment strictly follows the precautionary principle and strives for manure treatment plants that are emission-free. This principle is also funded by the Treaty on the Functioning of the European Union (2012/C 326/01), which strives towards environmental protection, and in turn, also affects public health [51]. Since the risks of bacterial or viral emissions of manure treatment plants to public health are still unclear, and thresholds for bioaerosol emissions do not yet exist, the “sealed shut principle” entails that any emissions have to be reduced in order to prevent possible public health risks to the greatest extent possible [52]. Furthermore, this province is involved in spatial planning, which considers health aspects. Consequently, large manure treatment plants need to be located in an industrial area, maintaining a minimum distance to residential areas. With regard to manure treatment, the province North Brabant has included a specific article in the environmental planning regulation that requires the guarantee of a healthy living environment. “Everything we do has to be associated to public health, on all policy areas.” (Interviewee 6). However, it is difficult to ensure a healthy living environment, due to many uncertainties regarding the public health risks of manure and manure treatment. “In accordance with the precautionary principle, we actually do not want any untreated manure applied on land, we only want treated manure.” (Interviewee 6).

### 3.2. Responsibility of Manure Related Public Health Protection

The interviews, as well as the system map, demonstrate that the legal system of manure handling, treatment and application, as well as public health policy, is highly fragmented and multiple authorities are involved. There is still much uncertainty surrounding the responsibilities regarding public health and manure. “It is a collective interconnected responsibility. There is no single player who is responsible for health. We have to collaborate.” (Interviewee 6). In line with this, the Ministry of VWS cannot take responsibility for all policy areas. “We assume that other ministries incorporate health into their considerations.” In addition, the Municipal Health Services (GGD) want to collaborate with all parties when a new policy is made. “We also want to include the citizens, action groups, as well as representatives of nature” (Interviewee 4). No interviewee reported a lack of cooperation. “There is rather overkill as you sit with so many parties at the table, which often reduces the discussions’ efficiency” (Interviewee 3). Another interviewee stated that “public health is insufficiently covered in the manure policy”. Moreover, Interviewee 4 stated that “you can reduce the health risks, when transport, treatment and protocol of emergencies are performed properly, and there are no emissions due to installations of air scrubbers. When there are no emissions, then you also do not have a public health risk. The farmer is primarily responsible for this. However, some farmers do not take this very seriously” (Interviewee 4). Therefore, the provinces and municipalities need to control whether farmers abide by the rules. The province North Brabant feels responsible for doing as much as possible for public health protection. Hence, they are involved in acquiring knowledge, contributing to discussions, and in sharing knowledge. “The starting point of all our actions is health” (Interviewee 6). Public health aspects can be incorporated in the licenses’ underlying regulations. The compliance with these regulations is monitored by municipalities, provinces, the Environmental Services, as well as the NVWA. “This is the role of the ministries. These would be the Ministry of VWS, the Ministry of LNV, as well as the Ministry of IenW. They should work together. The Ministry of VWS is responsible for health, whereas the Ministry of LNV has access to the measures” (Interviewee 6). Furthermore, there are research institutes and universities, which are responsible for the signalling of risks and, consequently, conduct research. Some interviewees said that nobody has yet been deemed responsible for manure and public health, whereas others said that the responsibility is fragmented. There was, however, consensus that the Ministry of VWS should be the responsible party. Additionally, Interviewee 2 affirms this. “Well, if there would be a problem, we would be the responsible party. But as long as there is no issue, we do not interfere”. As health is embedded in several policy domains, the Ministry of VWS does not have the sole responsibility. Even more so, the Ministry of VWS does not see manure of great importance for public health. “At least we do not have a known problem.” (Interviewee 2). To ensure sufficient protection, every authority and actor involved should include the public health aspects of manure handling and treatment, as well as its related regulations and policies. This does not only imply that farmers have to handle manure appropriately, but also that “parties that do not prioritize health, have to keep health in mind” (Interviewee 2). The incorporation of health into all sectors and levels of policy making is called the Health in All Policies approach (HiAP) [53]. As the policy makers at the Province of North Brabant have already stated, “health is prioritized in all our policy areas”. This could ensure that the possible risks of manure on public health can be further reduced.

## 4. Discussion

This study used interviews, as well as the literature, to produce a system map which depicts the Dutch manure governance and how public health aspects are considered in the Dutch manure policy. Public health protection aspects are indirectly covered in the Dutch manure policy. Primarily, the Dutch manure policies aim to protect environmental health, mainly water quality, from excess nutrients. The surplus of manure needs to be exported, for which manure first needs to undergo hygienisation. Manure treatment contributes to the microbial risk reduction of manure but is not performed to reduce the public health risks. Furthermore, different organizations are involved in the surveillance of zoonoses, which includes the signalling, assessing and control of zoonoses.

### 4.1. Constraints and Interrelationships

One of the main constraints for the adequate protection of public health and the environment is the complexity of the current manure policy. The manure policy is highly bureaucratic and the communication of its underlying facts and risks are not properly performed. Farmers experience the manure policies as being very fragmented and hard to understand [1]. This stresses the importance of communicating the strict manure regulations in an understandable manner. Besides, manure is not profitable for farmers, even though it is a valuable product. Farmers have to make the trade-off between the costs and returns of transporting manure. The complexity and high costs could have the consequence that farmers do not follow the regulations and commit fraud, which increases the environmental and public health problems [37]. This highlights the difficult position of farmers, who need to make a living, whilst being surrounded by strict regulations and being blamed for causing environmental pollution and health problems.

The interviews revealed another major constraint, which is the knowledge gap concerning the microbiological health risks of manure. Making policies to protect public health becomes difficult when the risks are unknown. This also complicates the process of granting permits. Due to these uncertainties, the precautionary principle is strictly applied, but it remains unclear whether this regulates the risks in an adequate manner. The Province of North Brabant also experiences these constraints, as their decisions on manure and public health cannot be substantiated, since this is still a grey area. Additionally, increased, proper research is needed in order to decide on the appropriate measures. In contrast, no signals of people getting infected of manure-borne pathogens have been reported. This, however, could be explained by the difficulty of tracing back the sources of infections, such as gastroenteritis, which only lasts a few days [54]. Since no signals of public health risks have reached the ministries, they are not concerned with exploring the need for additional actions regarding capacity building or policy implementation and enforcement.

This knowledge gap also distresses the residents who surround manure treatment plants. There is a shortage of manure treatment plants when compared to the manure surplus. However, the uncertainty on the public health risks forms an obstacle in the launch of new facilities due to residents’ protests against manure treatment plants. Still, residents suffer from odour nuisance and fear that these emissions can impair their health [13]. Besides residents’ opposition to intensive livestock production, manure fermenters present a risk of explosion, which causes even more concern [48,55]. As public health risks are unknown, these are difficult to communicate to the residents. This increases their distrust in science and the authorities, which in turn, increases their protest against any manure measures. The policy guideline on manure treatment plants and public health emphasizes the lack of scientific evidence of the public health risks emerging from manure and manure treatment [52].

### 4.2. Opportunities to Protect Public Health

Through adequate communication of the manure policy, farmers should be enabled to keep track of the complex system of manure without depending on consultancy offices. The extensive manure policy needs to be simplified and communicated adequately so that farmers are able to relate to its underlying reasons. Farmers play an important role in supporting environmental and public health protection. Raising awareness on the impacts of environmental and public health could be a further useful intervention. As farmers might have the highest risk of exposure to manure-borne pathogens themselves [56,57], raising awareness and promoting proper manure management and hygiene can reduce the potential risks.

A proper risk assessment is a fundamental step towards reducing possible infectious disease risk of manure and manure treatment. A quantitative microbial risk assessment (QMRA) can identify and quantify the risk when there is no epidemiological proof. This is crucial for filling this knowledge gap and can help in defining an acceptable level of risk of infection from manure-borne pathogens, which has not yet been determined. Since no quantitative risk assessment has confirmed the need for action, no regulations can be passed. Consequently, this can also alter the risk perception of residents when it is properly communicated. Risk communication is therefore another crucial aspect of the health risks of manure.

This stresses the importance of communication to not only farmers, but also to residents, who have to be taken into account. Being aware of residents’ concerns is fundamental in effective risk communication [58]. Communication can increase understanding of the farmer and the appreciation of their contribution to society, as well as restore the confidence that residents do not have towards authorities. Possibly, this could reduce the number of protests which hinder the construction and functioning of manure treatment plants. Especially when dealing with complex and uncertain risks such as manure, public involvement and communication is crucial in (risk) governance [59].

In broader terms, another opportunity to protect public health and improve the farmer’s situation could also be to discuss the role of CAP subsidies, which still fuel overproduction as well the problems associated with the manure surplus. Since the CAP intends to support farmers and ensure a sustainable food system, it should financially support farmers in the transition towards a sustainable agriculture. This could benefit the farmer, the environment, as well as public health [60,61]. One example, as was pointed out in the interviews, could entail supporting sustainable farm innovations for the storing and applying of manure that could reduce emissions and minimize adverse public health effects [62,63,64,65]. One possible approach entails farm systems which perform solid–liquid manure separation. Aside from reducing methane and ammonia emissions, as well as odour, to a great extent, separating these two forms of manure directly at the source might also reduce animal health risks. [66]. This promising innovation could also reduce the risk of zoonoses transmission to humans. In this case, increasing animal health, in turn, also benefits public health. In addition, many parties have stressed the benefits of manure hygienisation, which could have the potential to reduce the major microbiological risks [32,67]. Hygienisation and other forms of manure treatment, however, require manure treatment plants and suitable locations, which do not constitute a risk of a significant nuisance to residents.

Moreover, the Health in All Policies approach for public policies is important in the governance system of manure and public health. This concept rests on the idea that all policy areas that do not directly relate to health also affect health. Thus, all public policies should take health into account and avoid harmful impacts for public health [68].

### 4.3. Strengths & Limitations

This paper provides a comprehensive overview of the governance of manure and public health in an area of increased agricultural activity in the Netherlands, which is quite unique. Still, the applied system mapping method could also be used to uncover other issues regarding the environment and public health. The system map was based on seven interviews. We are aware of the limited number of interviewees. By including experts from other universities, ministries, the representatives of farmers, owners of manure treatment plants, as well as environmental services, this map could be enhanced. Extending the system map with different perspectives could give a more balanced and representative picture, and might provide more insights into policy recommendations. These findings represent the perspectives at the time of the interviews, which could be subject to change.

## 5. Conclusions

This article demonstrates how public health aspects are embedded in the Dutch manure policy. The large uncertainties on the potential risks of manure and manure treatment require a more stringent application of the precautionary principle. This complicates the decision-making for policymakers, who have to ensure a healthy environment and public health protection, but who also need to assess and grant permits for manure treatment plants to manage the manure surplus. Furthermore, this study highlights that the responsibilities for health protection with regards to manure are scattered. Many parties are involved in the complex system of manure and health, and public health benefits from other regulations which are intended, for instance, for animal health or environmental health protection. This highlights the importance of the concept of Health in All Policies, which means that public health protection should be incorporated across sectors, especially in complex systems such as manure policy and public health. Most importantly, this paper demonstrates the need for a health risk assessment of manure and manure treatment, which was identified to facilitate the authorisation of manure treatment plants and to mitigate health concerns. Such a health risk assessment can not only identify and quantify the risks, but also form a fundamental step towards adequate risk communication and intervention measures to protect public health.

## Figures and Tables

**Table 1:** Interviewees’ organizations and their roles.

#	Organization	Role
1	Netherlands Environmental Assessment Agency (PBL)	Policy researcher: Policy evaluations related to agriculture, environment and sustainability
2	Ministry of Health, Welfare and Sport (VWS)	Policymaker: zoonoses/communicable diseases
3	Ministry of Agriculture, Nature and Food Quality (LNV), team manure	Policymaker: manure policy
4	Municipal Health Service (GGD)	Environmental Health Scientist: manure (treatment) and health
5	Consultancy Health and Environment	Environmental Health Advisor
6	Province of North-Brabant	Policy maker: agrofood and living environment
7	Consultancy Sustainable Agriculture	Advisor sustainable agriculture

## Data Availability

Not applicable.

## Appendix A

Interview questions:

1. Please state your name and position
2. How does your work relate to manure and health?
3. What do you think about manure with regards to a healthy living environment?
4. What do you know about pathogens in manure?
5. What do you know about manure-borne pathogens in the environment?
6. Could you mention the health risks in relation to manure that you can think of? Do you see the relationship between manure and health in terms of infectious diseases?
7. Can you mention the existing regulations that consider manure (and health)?
8. Which regulations exist with regards to manure and manure treatment?
9. Are these regulations related to health?
10. In what way could the manure policy be improved so that public health aspects are considered?
11. Did you encounter any issues in the current manure regulations (regarding public health)?
12. Where in the system could health aspects with regards to manure be voiced?
13. Do you think that public health should be mentioned in manure regulations?
14. Who do you think is or should be responsible for manure and health (also regarding infectious diseases)?
15. Supposing manure poses a public health risk, which role could the Environmental and Planning Act play to reduce these effects?
16. Do you think there is a policy gap in relation to manure and public health?
17. Can you think of other actors that have a stake in manure and health?
18. Is there a need for more knowledge, cooperation, interventions within the system?
19. Do you think there is a solution for possible public health risks of manure?
20. Is there anything else you would like to say?
